# Non-Immersive Virtual Reality as an Intervention for Improving Hand Function and Functional Independence in Children With Unilateral Cerebral Palsy: A Feasibility Study

**DOI:** 10.7759/cureus.26085

**Published:** 2022-06-19

**Authors:** Chanan Goyal, Vishnu Vardhan, Waqar Naqvi

**Affiliations:** 1 Physiotherapy, Datta Meghe Institute of Medical Sciences, Wardha, IND; 2 Pediatric Physiotherapy, Government Physiotherapy College, Raipur, IND; 3 Research, N.K.P. Salve Institute of Medical Sciences and Research Center, Nagpur, IND

**Keywords:** rehabilitation, physiotherapy, motor learning, neuroplasticity, playstation, haptic feedback, virtual reality, non-immersive virtual reality, cerebral palsy

## Abstract

Introduction

Non-immersive virtual reality (NIVR) is emerging as an advantageous intervention in the arena of neurorehabilitation. Promising results have been obtained by the application of NIVR in adults with various chronic neurological conditions such as stroke and Parkinson’s disease, but studies on the use of NIVR in children with unilateral cerebral palsy (CP) are limited.

Materials and methods

This preliminary study included 10 school-aged participants with unilateral CP who were allocated into experimental and control groups. In accordance with the allocation ratio of 1:1, there were five participants in each group. During six weeks of intervention, children in the experimental group received NIVR intervention in addition to conventional physiotherapy, while those in the control group received only conventional physiotherapy, with a goal to improve hand function and functional independence. Nine-hole peg test (9HPT), box and block test (BBT), ABILHAND kids, and self-care section of functional independence measure for children (WeeFIM) were used as outcome measures.

Results

There was significant improvement in all outcome measures in both groups. However, the improvement in the hand function and functional independence was significantly more in the experimental group than in the control group.

Conclusion

It can be concluded that NIVR intervention in the management of children with unilateral CP seems to be feasible and useful. Further research with a larger sample size must be undertaken to reinforce these preliminary findings.

## Introduction

Non-immersive virtual reality (NIVR) is emerging as a means of intervention in the arena of neurorehabilitation. NIVR has been found to be beneficial in the rehabilitation of the geriatric population [1,2]. Promising results have been obtained by the application of NIVR in adult patients with varied health conditions such as stroke [3-7], Parkinson’s disease [8,9], and chronic obstructive pulmonary disease (COPD) [10], but studies on the use of NIVR in the pediatric population and specifically for children with unilateral cerebral palsy (CP) are limited [11].

Children with unilateral CP usually tend to avoid using the hand on the affected side, leading to dependence for activities that need bilateral hand usage. Consequently, there is increased burden of care on caregivers. This study aims to investigate the effect NIVR on hand function and functional independence in children with unilateral CP.

## Materials and methods

The study was conducted at Neurosciences Centre, Acharya Vinoba Bhave Rural Hospital, Datta Meghe Institute of Medical Sciences (DMIMS), Wardha, Maharashtra, India. The study was approved by the Institutional Ethical Committee of DMIMS with the approval number Ref.No. DMIMS(DU)/IEC/2020-21/131 and was executed in conformation to the Declaration of Helsinki. This pilot study is an interventional, non-randomized trial with an active control group. The inclusion criteria comprised age between 6 and 12 years, diagnosis of unilateral CP, levels I-III on Gross Motor Function Classification System (GMFCS), and levels I-III on Manual Ability Classification System (MACS). Exclusion criteria included epilepsy, surgery in the past six months, Botox treatment in the past three months, and inability to understand commands.

Screening for eligibility criteria was done. A parent or legal guardian of each participant signed the informed consent form. After the baseline assessment, this preliminary study recruited 10 participants with unilateral CP who were allocated into the experimental group (group A) and control group (group B). In accordance with an allocation ratio of 1:1, there were five participants in each group.

**Figure 1:**
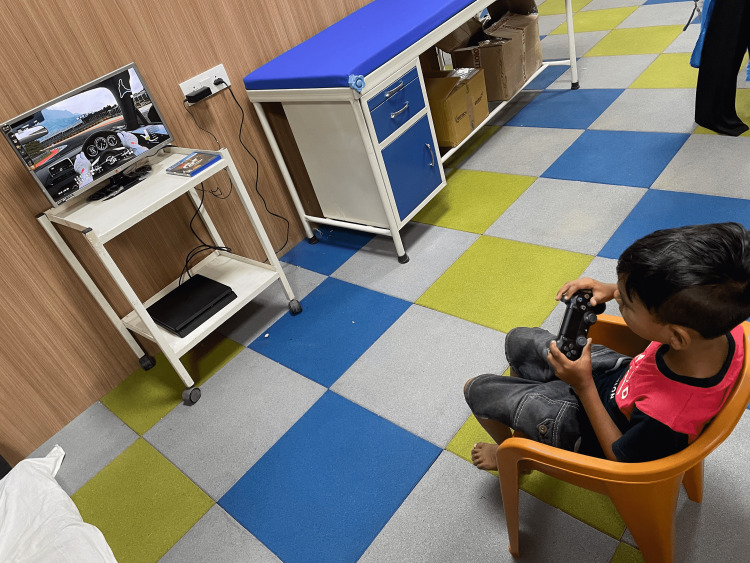
Non-immersive virtual reality based intervention

The duration of each session was 60 minutes. The children in the experimental group underwent 30 minutes of NIVR-based intervention using a driving simulation game with PlayStation 4 (Sony Interactive Entertainment Inc., Minato, Tokyo, Japan), as shown in Figure 1. In addition to NIVR, they also underwent 30 minutes of conventional physiotherapy that included weight-bearing exercises, multidirectional reaching activities, strengthening of weak muscles, and stretching of tight structures, while the children in the control group received 60 minutes of conventional physiotherapy for five days per week over a period of six weeks. Nine-hole peg test (9HPT) and box and block test (BBT) were used to evaluate hand function, whereas ABILHAND kids and self-care section of functional independence measure for children (WeeFIM) were used to measure functional independence. Pre- and post-intervention scores of all the outcome measures were analyzed and compared within groups and between groups.

## Results

**Figure 2:**
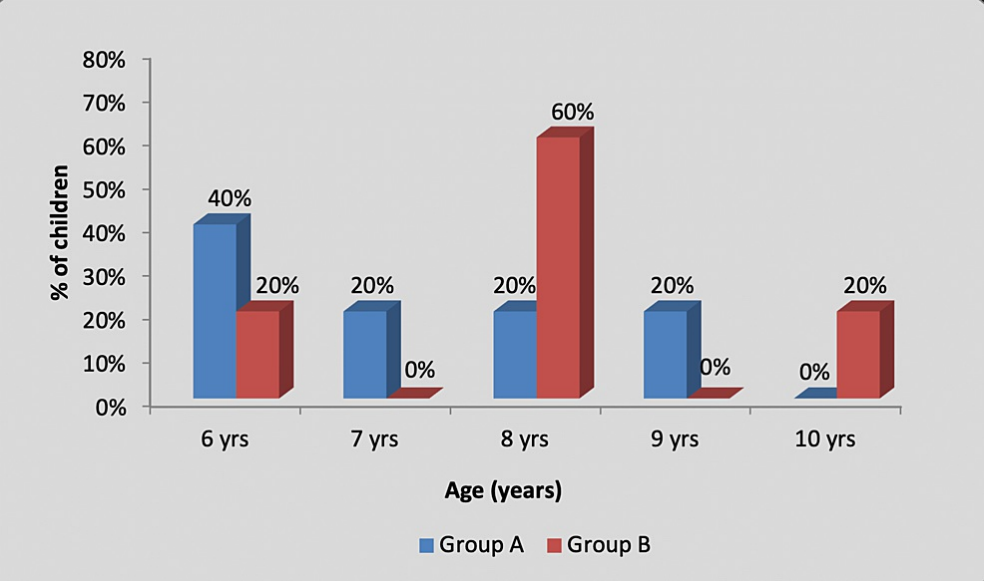
Graph showing age-wise distribution of children Yrs, years

There was no significant difference in the age-wise distribution of participants between group A (experimental) and group B (control), as shown in Table 1 and Figure 2.

**Table 1:** Age-wise distribution of children NS, non-significant; SD, standard deviation

Age (years)	Group A	Group B	X^2^ value
6	2 (40%)	1 (20%)	4.33, p=0.36, NS
7	1 (20%)	0 (0%)	
8	1 (20%)	3 (60%)	
9	1 (20%)	0 (0%)	
10	0 (0%)	1 (20%)	
Total	5 (100%)	5 (100%)	
Mean±SD	7.20±1.30	8±1.41	
Age range	6-9 years	6-10 years	

**Figure 3:**
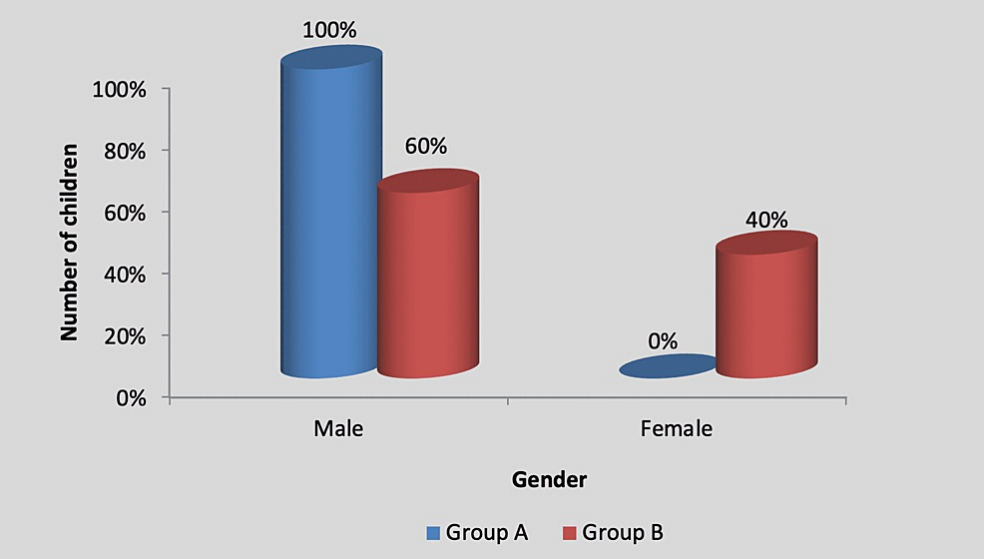
Graph showing gender-wise distribution of children

There was no significant difference in the gender-wise distribution of participants between group A (experimental) and group B (control), as shown in Table 2 and Figure 3.

**Table 2:** Gender-wise distribution of children NS, non-significant

Gender	Group A	Group B	X^2^ value
Male	5 (100%)	3 (60%)	2.50, p=0.15, NS
Female	0 (0%)	2 (40%)	
Total	5 (100%)	5 (100%)	

**Figure 4:**
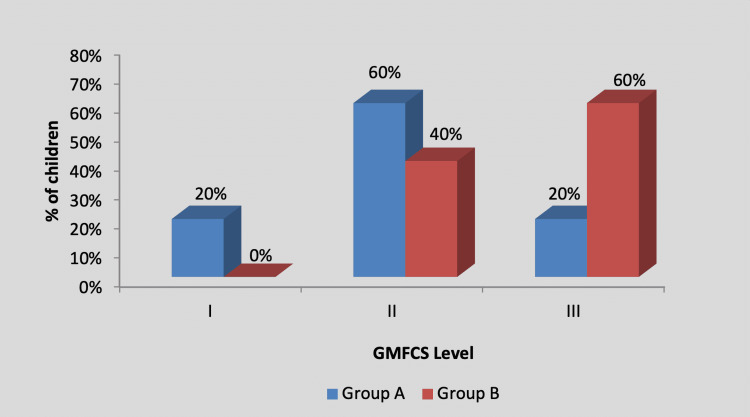
Graph showing distribution of children according to the GMFCS level GMFCS, Gross Motor Function Classification System

By using the chi-square test, statistically, no significant difference was found in the GMFCS level among participants in group A and group B (X2 value=2.20, p=0.33), as shown in Table 3 and Figure 4.

**Table 3:** Distribution of children according to the GMFCS level GMFCS, Gross Motor Function Classification System; NS, non-significant

GMFCS Level	Group A	Group B	X^2 ^value
I	1 (20%)	0 (0%)	2.20, p=0.33, NS
II	3 (60%)	2 (40%)	
III	1 (20%)	3 (60%)	
Total	5 (100%)	5 (100%)	

**Figure 5:**
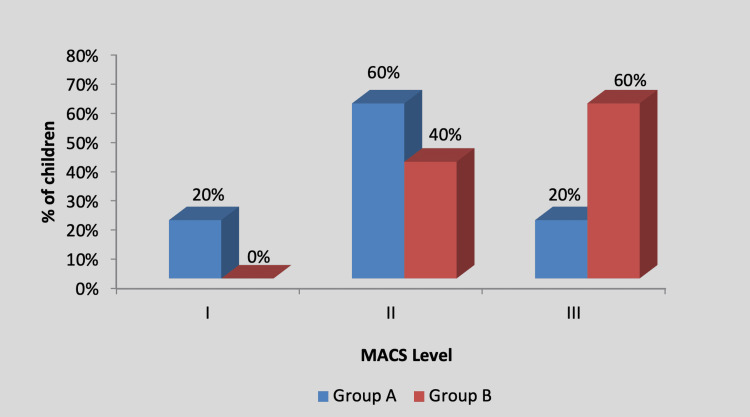
Graph showing distribution of children according to the MACS level MACS, Manual Ability Classification System

By using the chi-square test, no statistically significant difference was found in the MACS level among participants in group A and group B (X2 value=2.20, p=0.33), as shown in Table 4 and Figure 5.

**Table 4:** Distribution of children according to the MACS level MACS, Manual Ability Classification System; NS, non-significant

MACS level	Group A	Group B	X^2 ^value
I	1 (20%)	0 (0%)	2.20, p=0.33, NS
II	3 (60%)	2 (40%)	
III	1 (20%)	3 (60%)	
Total	5 (100%)	5 (100%)	

**Figure 6:**
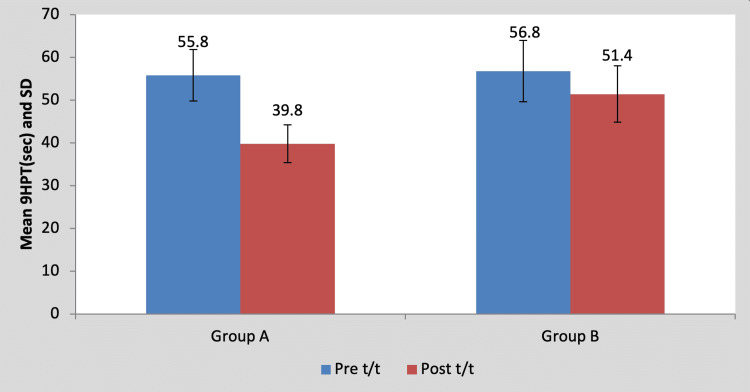
Graph showing comparison of pre- and post-treatment 9HPT scores in group A and group B 9HPT, nine-hole peg test; SD, standard deviation; t/t, treatment

Mean 9HPT score in the children of group A was 55.80±6.01 pre-treatment and it was 39.80±4.43 post-treatment. By using Student’s paired t-test, a statistically significant difference was found between pre-test and post-test 9HPT scores (t=16, p=0.0001), as shown in Table 5 and Figure 6. Mean 9HPT score in the children of group B was 56.80±7.19 pre-treatment and it was 51.40±6.58 post-treatment. By using Student’s paired t-test, a statistically significant difference was found between pre-test and post-test 9HPT scores (t=7.21, p=0.002), as shown in Table 5 and Figure 6.

**Table 5:** Comparison of pre- and post-treatment 9HPT scores in group A and group B by using Student’s paired t-test 9HPT, nine-hole peg test; S, significant; t/t, treatment

		Mean	N	Standard deviation	Standard error mean	Mean difference	t-Value
Group A	Pre t/t	55.80	5	6.01	2.69	16±2.23	16, p=0.0001, S
	Post t/t	39.80	5	4.43	1.98		
Group B	Pre t/t	56.80	5	7.19	3.21	5.40±1.67	7.21, p=0.002, S
	Post t/t	51.40	5	6.58	2.94		

**Figure 7:**
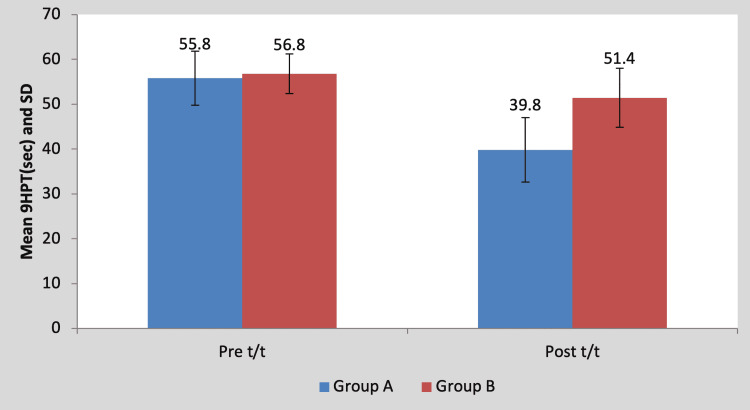
Graph showing comparison of pre- and post-treatment 9HPT scores between group A and group B 9HPT, nine-hole peg test; SD, standard deviation; t/t, treatment

Mean pre-treatment 9HPT score of the children in group A was 55.80±6.01 and in group B it was 56.80±7.19. By using Student’s unpaired t-test, no statistically significant difference was found in pre-treatment 9HPT scores between group A and group B (t=0.23, p=0.81), as shown in Table 6 and Figure 7. Mean post-treatment 9HPT score of the children in group A was 39.80±4.43 and in group B it was 51.40±6.58. By using Student’s unpaired t-test, a statistically significant difference was found in post-treatment 9HPT scores between group A and group B (t=3.26, p=0.011), as shown in Table 6 and Figure 7.

**Table 6:** Comparison of pre- and post-treatment 9HPT scores between group A and group B 9HPT, nine-hole peg test; NS, non-significant; S, significant

Test	Group A	Group B	t-value	p-value
Pre t/t	55.80±6.01	56.80±7.19	0.23	0.81, NS
Post t/t	39.80±4.43	51.40±6.58	3.26	0.011, S

**Figure 8:**
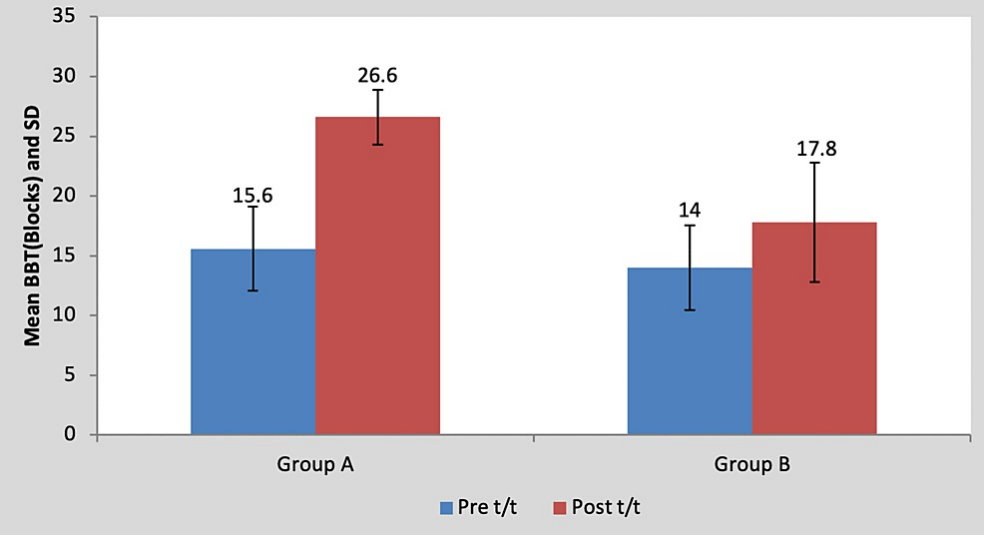
Graph showing comparison of BBT scores in group A and group B BBT, box and block test, S, significant; t/t, treatment

Mean BBT score in the children of group A was 15.60±3.50 pre-treatment and it was 26.60±2.30 post-treatment. By using Student’s paired t-test, a statistically significant difference was found between pre-test and post-test BBT scores (t=11.59, p=0.0001), as shown in Table 7 and Figure 8. Mean BBT score in the children of group B was 14±3.53 pre-treatment and it was 17.80±5.01 post-treatment. By using Student’s paired t-test, a statistically significant difference was found between pre-test and post-test BBT scores (t=5.17, p=0.0001), as shown in Table 7 and Figure 8.

**Table 7:** Comparison of pre- and post-treatment BBT scores in group A and group B by using Student’s paired t-test BBT, box and block test; S, significant; t/t, treatment

		Mean	N	Standard deviation	Standard error mean	Mean difference	t-Value
Group A	Pre t/t	15.60	5	3.50	1.56	11±2.12	11.59, p=0.0001, S
	Post t/t	26.60	5	2.30	1.02		
Group B	Pre t/t	14	5	3.53	1.58	3.80±1.64	5.17, p=0.0001, S
	Post t/t	17.80	5	5.01	2.24		

**Figure 9:**
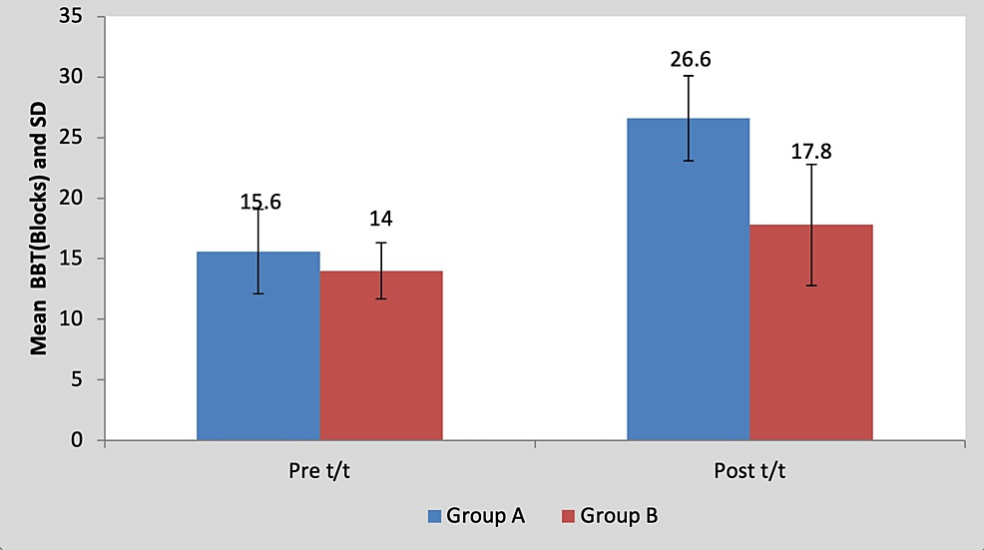
Graph showing comparison of BBT scores between group A and group B BBT, box and block test, SD, standard deviation; t/t, treatment

Mean pre-treatment BBT score of the children in group A was 15.60±3.50 and in group B it was 14±3.53. By using Student’s unpaired t-test, no statistically significant difference was found in pre-treatment BBT scores between group A and group B (t=0.71, p=0.49), as shown in Table 8 and Figure 9. Mean post-treatment BBT score of the children in group A was 26.60±2.30 and in group B it was 17.80±5.01. By using Student’s unpaired t-test, a statistically significant difference was found in post-treatment BBT scores between group A and group B (t=3.56, p=0.007), as shown in Table 8 and Figure 9.

**Table 8:** Comparison of BBT scores between group A and group B by using Student’s unpaired t-test NS, non-significant; S, significant; t/t, treatment

Test	Group A	Group B	t-Value	p-Value
Pre t/t	15.60±3.50	14±3.53	0.71	0.49, NS
Post t/t	26.60±2.30	17.80±5.01	3.56	0.007, S

**Figure 10:**
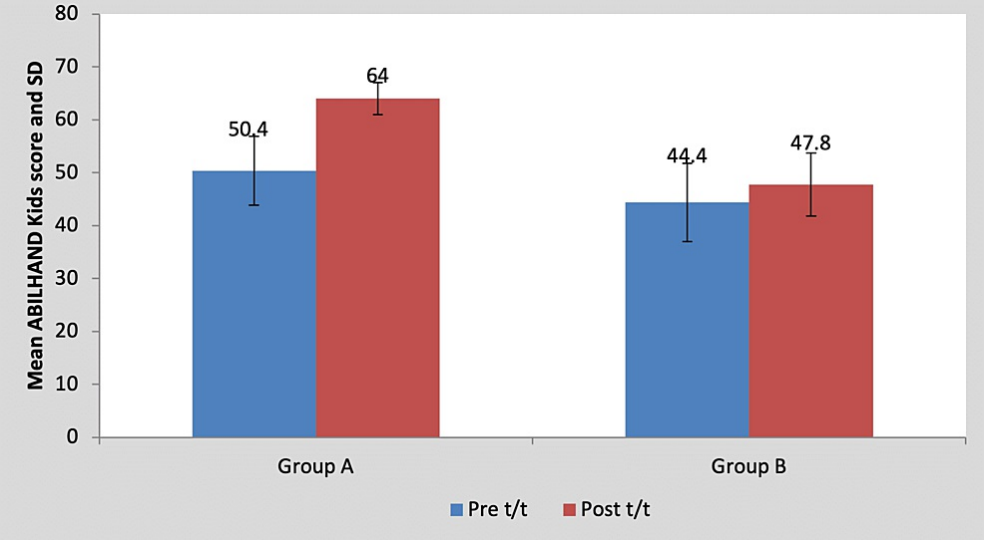
Graph showing comparison of ABILHAND Kids score in group A and group B SD, standard deviation; t/t, treatment

Mean ABILHAND kids score in the children of group A was 50.40±6.54 pre-treatment and it was 64.00±3.00 post-treatment. By using Student’s paired t-test, a statistically significant difference was found between pre-test and post-test ABILHAND kids scores (t=5.93, p=0.004), as shown in Table 9 and Figure 10. Mean ABILHAND kids score in the children of group B was 44.40±7.36 pre-treatment and it was 47.80±5.93 post-treatment. By using Student’s paired t-test, a statistically significant difference was found between pre-test and post-test ABILHAND kids scores (t=5.01, p=0.007), as shown in Table 9 and Figure 10.

**Table 9:** Comparison of ABILHAND Kids scores in group A and group B by using Student’s paired t-test S, significant; t/t, treatment

		Mean	N	Standard deviation	Standard error mean	Mean difference	t-Value
Group A	Pre t/t	50.40	5	6.54	2.92	13.60±5.12	5.93, p=0.004, S
	Post t/t	64.00	5	3.00	1.34		
Group B	Pre t/t	44.40	5	7.36	3.29	3.40±1.51	5.01, p=0.007, S
	Post t/t	47.80	5	5.93	2.65		

**Figure 11:**
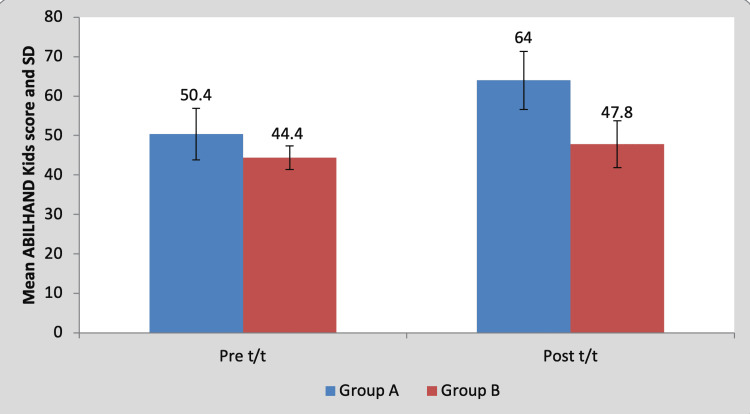
Graph showing comparison of ABILHAND Kids scores between group A and group B SD, standard deviation; t/t, treatment

Mean pre-treatment ABILHAND kids score of the children in group A was 50.40±6.54 and in group B it was 44.40±7.36. By using Student’s unpaired t-test, no statistically significant difference was found in pre-treatment ABILHAND kids scores between group A and group B (t=1.36, p=0.21), as shown in Table 10 and Figure 11. Mean post-treatment ABILHAND kids score of the children in group A was 64±3 and in group B it was 47.80±5.93. By using Student’s unpaired t-test, a statistically significant difference was found in post-treatment ABILHAND kids scores between group A and group B (t=5.44, p=0.001), as shown in Table 10 and Figure 11.

**Table 10:** Comparison of ABILHAND Kids scores between group A and group B by using Student’s unpaired t-test NS, non-significant; S, significant; t/t, treatment

Test	Group A	Group B	t-value	p-value
Pre t/t	50.40±6.54	44.40±7.36	1.36	0.21, NS
Post t/t	64±3	47.80±5.93	5.44	0.001, S

**Figure 12:**
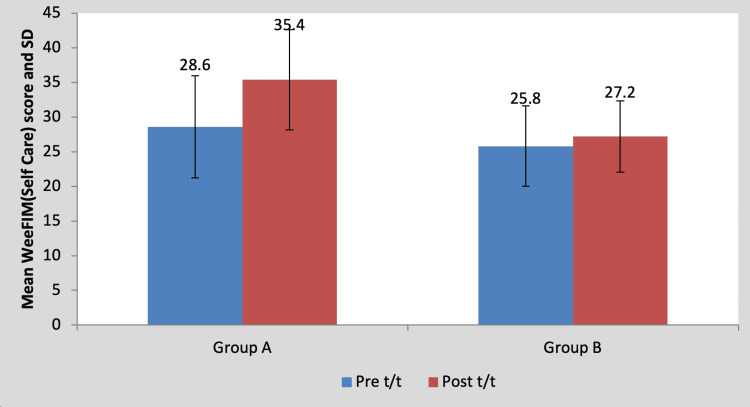
Graph showing comparison of WeeFIM (self-care) scores in group A and group B FIM, functional independence measure; SD, standard deviation; t/t, treatment

Mean WeeFIM (self-care) score in the children of group A was 28.60±7.36 pre-treatment and it was 35.40±7.23 post-treatment. By using the Wilcoxon signed rank a test, a statistically significant difference was found between pre-test and post-test WeeFIM (self-care) scores (z=18.17, p=0.0001), as shown in Table 11 and Figure 12. Mean WeeFIM (self-care) score in the children of group B was 25.80±5.80 pre-treatment and it was 27.20±5.16 post-treatment. By using the Wilcoxon signed rank test, a statistically significant difference was found between pre-test and post-test WeeFIM (self-care) scores (z=2.76, p=0.042), as shown in Table 11 and Figure 12.

**Table 11:** Comparison of WeeFIM (self-care) scores in group A and group B by using the Wilcoxon signed rank test S, significant; t/t, treatment

		Mean	N	Standard deviation	Standard error mean	Mean difference	z-Value
Group A	Pre t/t	28.60	5	7.36	3.295	6.80±0.83	18.17, p=0.0001, S
	Post t/t	35.40	5	7.23	3.23		
Group B	Pre t/t	25.80	5	5.80	2.59	1.40±1.14	2.76, p=0.042, S
	Post t/t	27.20	5	5.16	2.31		

**Figure 13:**
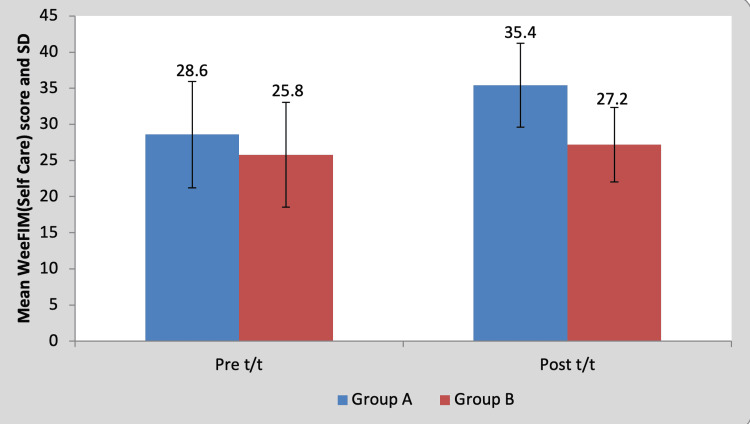
Graph showing comparison of WeeFIM (self-care) scores between group A and group B FIM, functional independence measure; SD, standard deviation; t/t, treatment

Mean pre-treatment WeeFIM (self-care) score of the children in group A was 28.60±7.36 and in group B it was 25.80±5.80. By using the Mann-Whitney U test, no statistically significant difference was found in pre-treatment WeeFIM (self-care) scores between group A and group B (t=0.66, p=0.52), as shown in Table 12 and Figure 13. Mean post-treatment WeeFIM (self-care) score of the children in group A was 35.40±7.23 and in group B it was 27.20±5.16. By using the Mann-Whitney U test, a statistically significant difference was found in post-treatment WeeFIM (self-care) scores between group A and group B (t=2.56, p=0.042), as shown in Table 12 and Figure 13.

**Table 12:** Comparison of WeeFIM (self-care) scores between group A and group B by using the Mann-Whitney U test NS, non-significant; S, significant; t/t, treatment

Test	Group A	Group B	t-value	p-value
Pre t/t	28.60±7.36	25.80±5.80	0.66	0.52, NS
Post t/t	35.40±7.23	27.20±5.16	2.56	0.042, S

Statistical analysis was conducted by descriptive and inferential statistics using the chi-square test, Student’s paired and unpaired test, Wilcoxon signed rank test, and Mann-Whitney U test, software used in the analysis were SPSS 27.0 version (IBM Corp., Armonk, NY, USA) and GraphPad Prism 7.0 version, and p<0.05 was considered as a level of significance.

## Discussion

The protocol of this study has been adapted from a previously published study [12], though the sample size is limited as this is a pilot study. Similar to the findings of the present study, previous studies supported the possibility of using NIVR as an intervention for children with hemiplegic CP who avoid using the hand on the affected side [13-15]. In 2019, Martins et al. concluded that practice of tasks in virtual environment helped in performing the real tasks [13]. Also, gait and gross motor function has shown improvement with NIVR intervention in children with CP [16].

The principles of neuroplasticity and that of motor learning including explicit feedback and multimodal stimulation are well tapped by virtual reality (VR) systems [17]. Besides, conventional physiotherapy in the form of active exercises performed by the participants in both groups must have contributed in bringing about the positive changes [18]. All the outcome measures used in the study, namely, 9HPT [19], BBT [20], ABILHAND kids [21], and WeeFIM [22], are valid and reliable. Apart from statistically significant differences noted between the experimental and control groups, clinically significant difference was observed in hand function and functional independence between both groups.

Immersive VR systems use head-mounted display [23] that may not be tolerated well by young children between 6 and 12 years of age [24]. NIVR-based intervention was reported to be comfortable and enjoyable by the children. No negative effect of NIVR intervention was noted during the study, similar to what was remarked in a systematic review published in 2020 [25]. The children were well-engaged during the NIVR sessions. They were intrinsically motivated to actively use both hands for gaming.

Apart from neurorehabilitation, VR has been utilized as a distraction for pain management in children [26,27]. VR-based games have also been investigated as a tool for telerehabilitation [28]. There is a plethora of systems that provide options for NIVR gaming. Nevertheless, further research on innovative applications of this user-friendly approach is warranted.

## Conclusions

It can be concluded that the study design is feasible and can be used with a larger sample size for further trial. The preliminary findings of this study, although limited by a small size of the sample, indicate that NIVR deserves exploration as a viable intervention for improving hand function and for decreasing dependence in the performance of routine activities for children with unilateral CP. NIVR came out as an interesting way to engage children with unilateral CP in an activity that requires bilateral hand use, which they otherwise avoided.
